# InteractiVenn: a web-based tool for the analysis of sets through Venn diagrams

**DOI:** 10.1186/s12859-015-0611-3

**Published:** 2015-05-22

**Authors:** Henry Heberle, Gabriela Vaz Meirelles, Felipe R da Silva, Guilherme P Telles, Rosane Minghim

**Affiliations:** Universidade de São Paulo, Instituto de Ciências Matemáticas e de Computação, Av. Trabalhador São-carlense, 400, São Carlos SP, Brazil; Laboratório Nacional de Biociências, Campinas SP, Caixa Postal 6192 Brazil; Embrapa Informática Agropecuária, Av. André Tosello, 209, Campinas SP, Brazil; Universidade Estadual de Campinas, Instituto de Computação, Av. Albert Einstein, 1251, Campinas SP, Brazil

**Keywords:** Venn diagram, Edwards-Venn diagram, Interaction, Set-unions

## Abstract

**Background:**

Set comparisons permeate a large number of data analysis workflows, in particular workflows in biological sciences. Venn diagrams are frequently employed for such analysis but current tools are limited.

**Results:**

We have developed InteractiVenn, a more flexible tool for interacting with Venn diagrams including up to six sets. It offers a clean interface for Venn diagram construction and enables analysis of set unions while preserving the shape of the diagram. Set unions are useful to reveal differences and similarities among sets and may be guided in our tool by a tree or by a list of set unions. The tool also allows obtaining subsets’ elements, saving and loading sets for further analyses, and exporting the diagram in vector and image formats. InteractiVenn has been used to analyze two biological datasets, but it may serve set analysis in a broad range of domains.

**Conclusions:**

InteractiVenn allows set unions in Venn diagrams to be explored thoroughly, by consequence extending the ability to analyze combinations of sets with additional observations, yielded by novel interactions between joined sets. InteractiVenn is freely available online at: www.interactivenn.net.

## Background

In biological sciences it is often necessary to compare sets of data such as genes, proteins, organisms as well as other entities. The same set comparison activities can be useful to many other research fields. Sets and their unions and intersections may be conveniently displayed as Venn diagrams, a widely adopted and familiar layout [1]. A recent example can be seen in the article describing the banana genome [2], where a Venn diagram illustrates the relationship among six plant genomes. Venn diagrams may also be used as a means to explore and reason about data, but most often such diagrams provide only static views of up to four sets of data.

Although Venn diagrams may be built for any number of sets, the layout becomes increasingly challenging beyond four sets. For instance, we can use circles to create a symmetric three-set diagram but we cannot use them with four or more sets and still show all possible intersections and exclusive regions. The same holds for ellipses: we cannot create a diagram with more than five sets with them. Many other shapes may be used, such as squares, triangles and spherical surface segments, the latter being a widely adopted layout proposed by Edwards [1]. A Venn diagram could even be formed by elements with abstract shapes, but not without difficulties in showing all necessary regions and in generalizing for any number of sets.

With a larger number of sets, symmetric Venn diagrams are easier to interpret because the reader needs less time to locate regions of interest and their boundaries, as well as set intersections. Considering restrictions in aesthetics and burden on human visual ability, Venn diagrams of seven sets just seem to be too much.

Many websites and graphics programs allow users to manually draw labeled Venn diagrams. Other programs extend the basic drawing, automating the construction of diagrams from lists of elements in each set. Examples include Pangloss Venn diagram generator (http://www.pangloss.com/seidel/Protocols/venn4.cgi) and Venny (http://bioinfogp.cnb.csic.es/tools/venny/), both restricted to static diagrams of up to four sets. Other examples are DrawVenn (http://bioinformatics.psb.ugent.be/webtools/Venn/) and BioVenn [3] that construct area-proportional diagrams, limited by the fact that it is not possible to create symmetric Venn diagrams with more than four sets that respect proportionality. Software systems that build area-proportional diagrams for larger number of sets use heuristics and typically produce Euler diagrams without support for all possible intersections among sets.

A few programs extend the functionality beyond drawing the diagram and listing the elements in each diagram region. GeneVenn [4] and VennMaster [5] link genes in each set to Entrez (www.ncbi.nlm.nih.gov) or to Gene Ontology (www.geneontology.org). jvenn [6] is a Javascript library and viewer that enables to compare up to six lists. Vennture [7] improves data integration of sets comparison. GeneSpring (http://www.genomics.agilent.com/en/product.jsp?cid=cat170014) integrate Venn diagrams into the analysis of microarray data. This is also the case of SilicoCyte (http://www.selectscience.net/products/silicocyte/?prodid=11193).

However, these choices are limited either in the number of sets they handle or in the ability to display partial set operations (such as partial unions inside the diagram). Displaying partial unions, that is, locating regions that combine unions of sets and their intersections, and highlighting such regions interactively, can provide additional insight to the analyst. In this article we describe InteractiVenn, a web-based tool to build and analyze Venn diagrams. InteractiVenn provides the ability to interactively visualize the effect of successive unions of up to six sets, enhancing the user capacity of analyzing data. Additionally, set unions are allowed in any order, including along a union list or a binary tree, enabling different perspectives on the data, for instance in the analysis of sets of genes across species related by a phylogeny.

## Implementation

InteractiVenn was implemented as a web application in HTML and Javascript, using functions provided by libraries D3.js (http://d3js.org), jPaq (http://jpaq.org), JSColor (http://jscolor.com) and FileSaver (http://github.com/eligrey/FileSaver.js).

## Results and discussion

**Figure 1:**
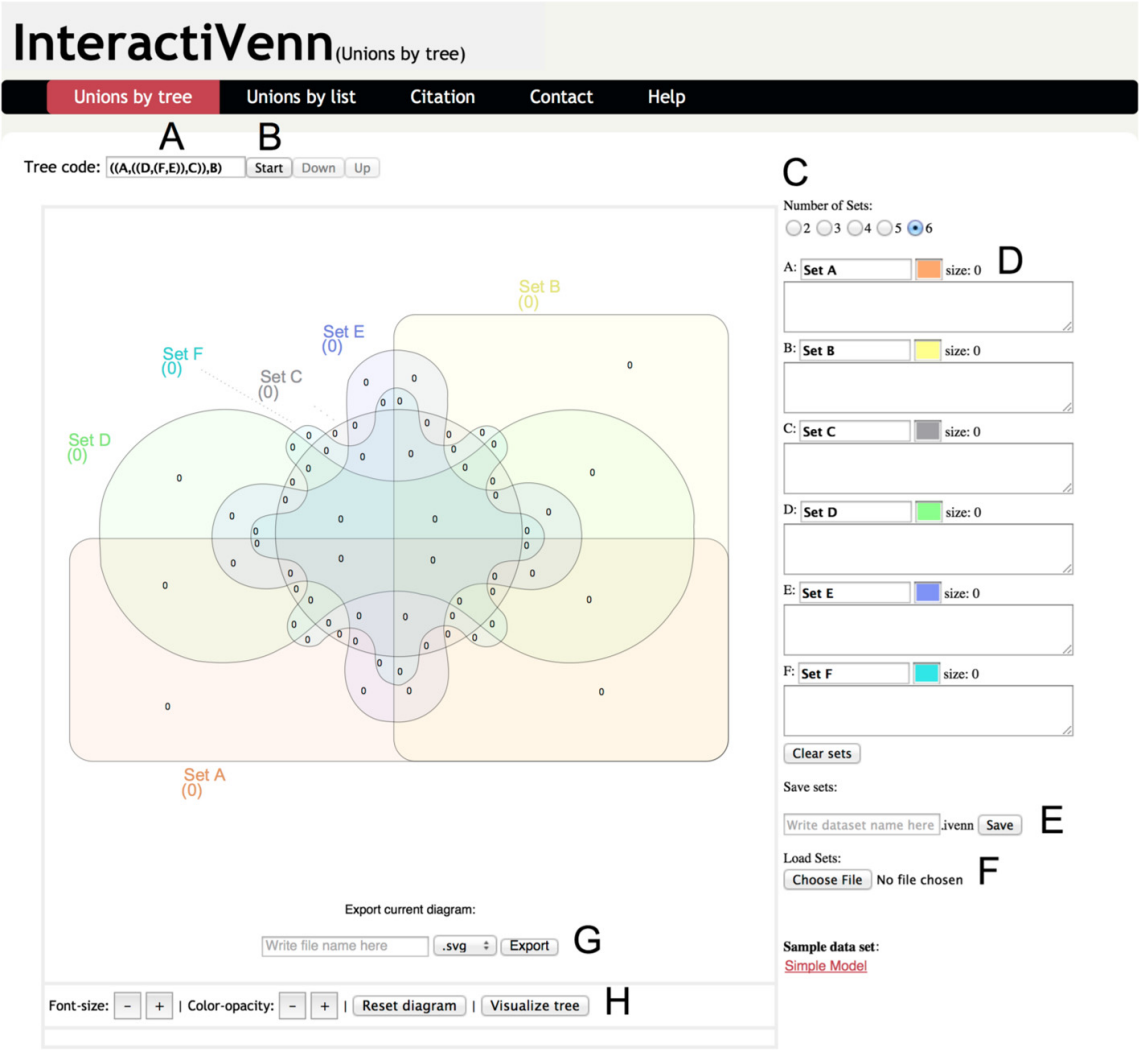
InteractiVenn Interface. InteractiVenn interface’s main parts are indicated by lettering as follows: (**A**) unions sequence; (**B**) the button that starts the sequence of set unions defined in (**A**); (**C**) the selector of the number of sets of the diagram; (**D**) fields to be filled with the elements of each set, one element (string) per line; (**E**) controls to download the current sets as a text file; (**F**) controls to upload sets previously saved; (**G**) controls to export the current diagram with extension in SVG format and (**H**) controls to increase and decrease font size and color opacity, to reset the diagram’s colors and font and to display the tree that will guide unions.

InteractiVenn offers a clean interface, as shown in Fig. 1. To build a diagram, the user starts selecting the number of sets, which also determines the diagram shape. The user may type or paste the name of each set, as well as its elements (Fig. 1D). Each element of a set is a string terminated by the end of a line. The diagram updates as sets are filled. The sets can also be loaded from a file.

When the user moves the cursor over the diagram, the set under the cursor will be highlighted to help identify its intersections. Transparency is employed to help locate intersections and ease the identification of each region in the diagram. Pressing a mouse button on a non-empty region triggers the display of a list of the elements in the intersection of sets represented by that region.

InteractiVenn enables unions of sets preserving the diagram shape. Such unions of sets are often useful to unveil differences and similarities among them. The unions may be guided either by a binary tree or by a list. The selection between tree or list is made via the main toolbar of InteractiVenn.

**Figure 2:**
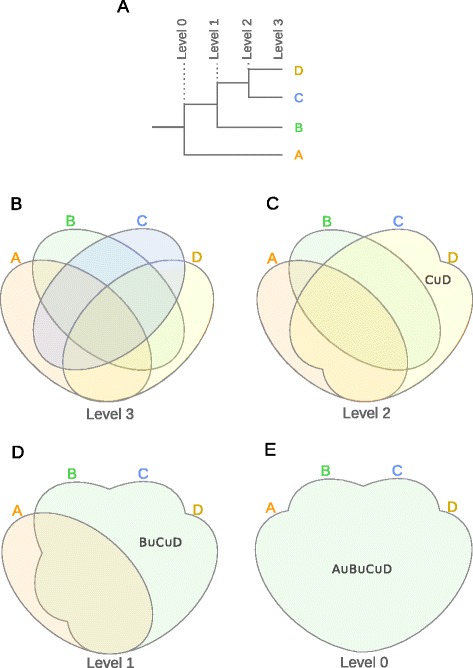
Venn diagram construction by a sequence of union operations. (**A**) binary tree; (**B**) Venn diagram for level 3; (**C**) Venn diagram for level 2; (**D**) Venn diagram for level 1; (**E**) Venn diagram for level 0.

A sequence of unions guided by a tree is shown in Fig. 2. The depicted tree can be coded as (*A*,(*B*,(*C*,*D*))), where parentheses group subtrees and commas indicate bifurcation (following Newick tree format (http://evolution.genetics.washington.edu/phylip/newick_doc.html)). With (*A*,(*B*,(*C*,*D*))) typed in the appropriate field in InteractiVenn’s interface, the user may navigate across tree levels, starting from the leaves level, where sets *A*,*B*,*C* and *D* are distinct. Navigating up the tree will render the diagram for sets *A*, *B*, *C*∪*D*, then the diagram for *A* and *B*∪*C*∪*D*, and finally the diagram for *A*∪*B*∪*C*∪*D*. The diagram will be updated to reflect the size of each region, subject to diagram unions. Navigating through diagrams will thus allow the user to inspect the effect of unions instantly. For instance, going from level 2 to level 1 in Fig. 2 allows to observe the intersections among *A*, *B* and the common ancestor of *C* and *D*. The tree itself is also shown by InteractiVenn.

A more general union specification may be given by a list of sets to unite at each step. The navigation between diagrams obtained through the tree in Fig. 2 may be also obtained specifying the list ;*CD*;*CDB*,*CDBA*. The list allows defining unions that are not hierarchically related, for instance ;*CD*;*CB*;*AB*,*CD* will produce a diagram where no sets are united, then one diagram with the sets *A*, *B* and *C*∪*D*, another diagram with *A*, *C*∪*B* and *D*, and finally a diagram with the sets *A*∪*B* and *C*∪*D*.

InteractiVenn allows adjusting font size as well as setting and opacity, that may be used by the user to tune the diagram layout. It also allows exporting the diagram in PNG and SVG formats, and the elements in every diagram region may be exported as a text file. InteractiVenn allows saving the sets to a local file that can be uploaded in a future moment for further analyses.

### Study case 1: comparing feature selection methods for candidate biomarkers discovery

To show the usefulness of our tool, we have analyzed a published prostate cancer proteomic dataset [8], searching for candidate biomarkers through feature selection analyses. In a previous work by Kawahara *et al.* [9], a discovery-to-target pipeline was proposed to analyze proteomics data, comprising a mass spectrometry (MS)-based discovery, three feature selection methods, clustering, Venn diagram, bioinformatics analyses and targeted approaches. The feature selection methods used in the pipeline were the univariate Beta-binomial [10], the semi-multivariate Nearest Shrunken Centroids (NSC) [11] and the multivariate Support Vector Machine-Recursive Features Elimination (SVM-RFE) [12]. The proof-of-concept was performed in a well-controlled proteomic data from the secretomes of three human cell lines, and was also validated on the published prostate cancer proteomic dataset [8].

Here, in order to generate lists of proteins sorted by relevance in discriminating the two classes in the dataset (organ-confined (OC) and extracapsullar (EC) prostate cancer cells), five methods were used, including the three used before in the discovery-to-target pipeline [9], the classical t test and the MWW test, all implemented in R. These and many other methods are being developed and applied for this type of study and they need to be compared for a deeper understanding on the distribution of candidate biomarkers resulting from the different methods.

As described by Tibshirani *et al.* [11], we may consider the top-n proteins to look for potential biomarkers. Although there are statistical validation procedures to define the value of n and to calculate false-positive and false-negative rates for each method [13], we adopt the vision that it is also important to compare all resulting lists of proteins, since those that appear in most methods may be more reliable or may lead to less false-positives, and thus can be further used by biologists in future experiments. Moreover, in a second analysis step, we may compare both the intersecting and exclusive proteins of each method to determine if one is good for potential biomarkers identification.

**Figure 3:**
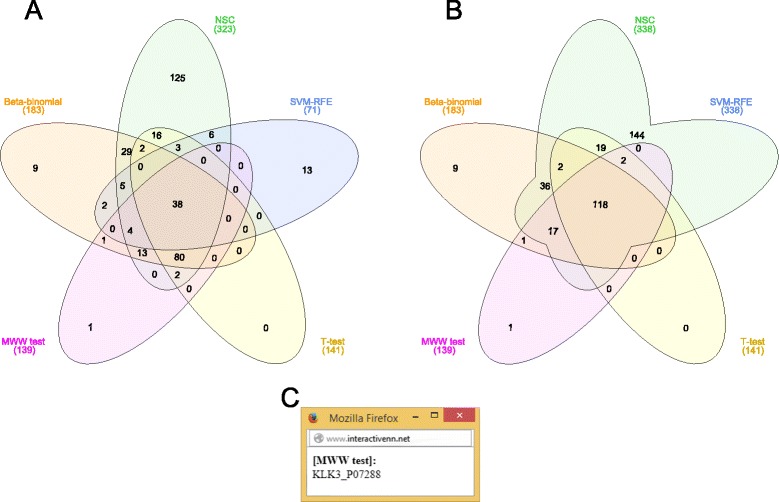
Comparison of ranked lists of candidate biomarkers by five feature selection methods. (**A**) 38 proteins are shared by all methods, whereas the semi and multivariate methods show more exclusive proteins than the univariate ones; (**B**) 144 proteins are exclusively shared by the semi and multivariate methods; (**C**) KLK3 was retrieved as an exclusive protein by only the MWW test.

Based on the confidence level (p-value ≤0.05) for the univariate methods (Beta-binomial, t test and MWW test) and on the double cross-validation procedure for the semi and multivariate methods (NSC and SVM-RFE, respectively), the top-n final ranked lists of candidate biomarkers resulting from each model were compared. In total, all five methods have shown 349 different proteins (union code: *ABCDE*). Figure 3A shows that all methods retrieved 38 common proteins, while the semi and multivariate methods have, in general, more exclusive proteins than the univariate ones. We can also see that the semi and multivariate methods exclusively share 6 proteins, whereas among the univariate methods, only one protein is exclusively shared by the Beta-binomial and MWW tests. Union operations allow us to see different patterns, for instance, by using the code BC to trigger the union of sets B (NSC) and C (SVM-RFE), we see that 144 proteins were retrived exclusively by the semi and multivariate methods (Fig. 3B). The approach adopted by InteractiVenn preserves the position and shape of the sets, allowing a smoother exploration. Other unions are possible as well.

Furthermore, seven proteins identified as candidate biomarkers in the prostate cancer cells in the work by Kim *et al.* [8] were also verified in the same work by experimental biochemical methods and were searched in the Venn diagram sets built using the InteractiVenn tool: KLK3 (PSA), ACPP (PAP), SFN, MME, PARK7, TIMP1 e TGM4. Notably, from these proteins, KLK3 was the only one not validated as a candidate biomarker and, using InteractiVenn, we could observe that it was retrieved as an exclusive protein only by the MWW test (Fig. 3C). Out of the other six validated candidates, four (ACPP, SFN, MME e TGM4) were found in the intersection among the three methods used in the discovery-to-target pipeline [9], one (PARK7) was found in the intersection between Beta-binomial and NSC, and another one (TIMP1), in the intersection between NSC and SVM-RFE. Interestingly, none was found exclusively by the t test, suggesting that the three methods used in the pipeline described by Kawahara *et al.* [9] could retrieve the best potential candidate biomarkers in their intersections.

### Study case 2: distribution of gene families among six plant genomes

A Venn diagram comparing six plant proteomes is shown in the article describing the banana (*Musa acuminata*) genome and the evolution of monocotyledonous plants [2]. The diagram shows the distribution of shared gene families (sequence clusters) among the proteomes of five monocotyledons (*Musa acuminata*, *Phoenix dactylifera*, *Oryza sativa*, *Sorghum bicolor* and *Brachypodium distachyon*) and one eudicotyledon (*Arabidopsis thaliana*), and it was used to compare how many sequence clusters exist in common among these species.

**Figure 4:**
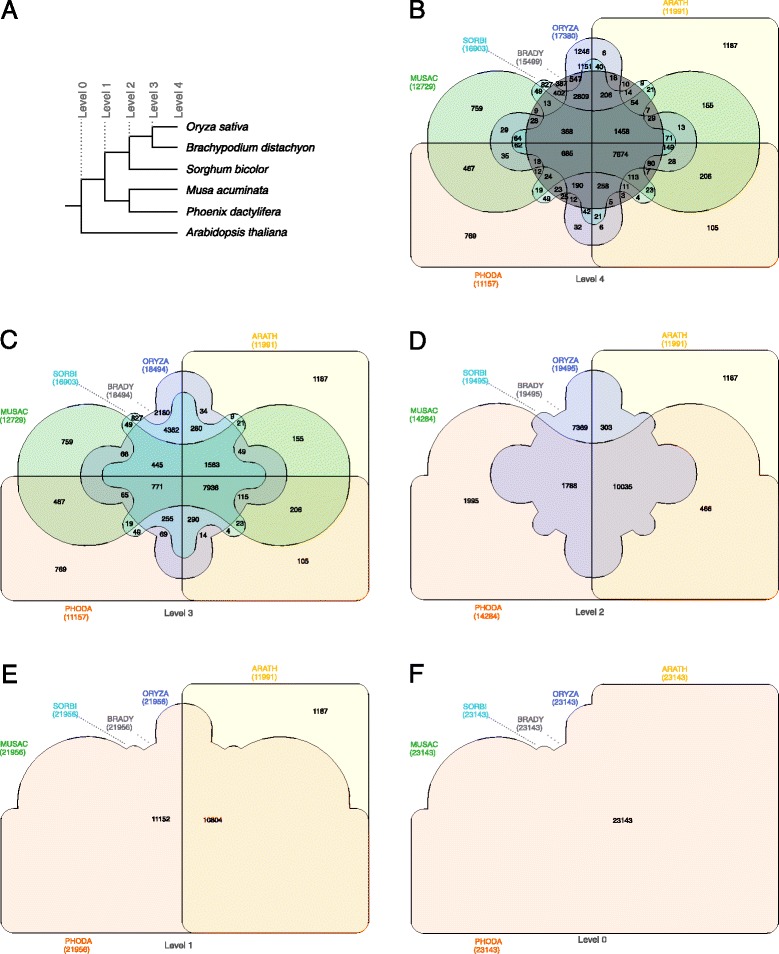
Venn diagram showing the distribution of shared gene families (sequence clusters) among six plant proteomes. The Venn diagram was constructed by a sequence of union operations following the hierarchy of a binary tree based on the work by D’Hont *et al.* [2]. (**A**) binary tree; (**B**) Venn diagram for level 4; (**C**) Venn diagram for level 3; (**D**) Venn diagram for level 2; (**E**) Venn diagram for level 1; (**F**) Venn diagram for level 0. ORYZA: *Oryza sativa*; BRADY: *Brachypodium distachyon*; SORBI: *Sorghum bicolor*; MUSAC: *Musa acuminata*; PHODA: *Phoenix dactylifera*; ARATH: *Arabidopsis thaliana*.

The data set of the *M. acuminata* article was converted to the InteractiVenn format to reproduce the Venn diagram described above. The result is shown in Fig. 4B. The *M. acuminata* article also presents a binary tree showing the timing of whole-genome duplications relative to speciation events within representative monocotyledons and eudicotyledons. The phylogenetic tree representing the relations between the different plant species could also be used to relate information between the sets in a Venn diagram. Set unions as provided by InteractiVenn allow the identification of similarities and differences between the species groups, as defined by the tree hierarchy.

As an example, Fig. 4A shows a simplified tree that represents the hierarchy that appears in the *M. acuminata* article, with only six species. Starting the union operations according to this tree, we have at level 0 the union of all sets (Fig. 4F). Following the hierarchy, InteractiVenn then groups all monocotyledons at level 1, because at this level they are in the same branch (Fig. 4E). It is possible to identify unique sequence clusters of monocotyledons relative to *Arabidopsis thaliana*. Navigating to level 2 (Fig. 4D), the diagram shows two groups between the monocotyledons: the union of *O. sativa*, *B. distachyon* and *S. bicolor*, and the union of *M. acuminata* and *P. dactylifera*. At level 3 (Fig. 4C) there is only one group: *O. sativa* and *S. bicolor*. When level 4 is reached, the diagram shows each species’ set individually (Fig. 4B).

## Conclusions

More than providing a wider set of features to publish results of sets comparisons, InteractiVenn extends the ability to analyze combinations of sets of elements in part or in total, affording additional observations on the interactions between joined sets. We expect that InteractiVenn will be used by biologists in a wide range of tasks and also by other researchers who seek for more flexible control of the examination of set unions in Venn diagrams of objects.

## Availability and requirements

**Project name**: InteractiVenn**Project home page**: http://www.interactivenn.net/**Operating system**: Platform independent**Programming language**: Javascript and HTML**Other requirements**: Web browsers Firefox 37 or later or Chrome 42 or later**License**: GPLv3**Any restrictions to use by non-academics**: none
